# Strategies to Increase Filipino American Participation in Cardiovascular Health Promotion: A Systematic Review

**DOI:** 10.5888/pcd15.170294

**Published:** 2018-05-17

**Authors:** Jermy-Leigh B. Domingo, Gretchenjan Gavero, Kathryn L. Braun

**Affiliations:** 1 Hawai‘i Primary Care Association, Honolulu, Hawai‘i; 2 University of Hawai‘i John A. Burns School of Medicine, Department of Psychiatry, Honolulu, Hawai‘i; 3 University of Hawai‘i at Mānoa, Office of Public Health Studies, Honolulu, Hawai‘i

## Abstract

**Introduction:**

Cultural tailoring of interventions can be effective in reducing health disparities by attracting underserved populations to health promotion programs and improving their outcomes. The purpose of this systematic review was to assess what is known about increasing access to and participation in cardiovascular disease (CVD) prevention and control programs among Filipino Americans.

**Methods:**

PubMed MEDLINE, CINAHL, and Sociologic Abstracts were searched for peer-reviewed studies and dissertations conducted in the United States from 2004 through 2016.

**Results:**

A total of 347 articles were identified through the search, and 9 articles reporting on 7 interventions focused on CVD prevention in a Filipino American sample were included. All but one intervention used evidence-based curricula, and implementation varied across sites. All but 2 interventions used word-of-mouth advertising from friends, family, and community leaders to increase participation. The Filipino cultural values of food, social relationships, and family were prevalent aspects across interventions tailored for Filipino Americans. Aspects of spirituality and the arts were integrated into only 3 studies.

**Conclusion:**

Given the burden of CVD in Filipino American populations, tailored interventions rooted in Filipino cultural values are vital to address this known health disparity.

## Introduction

Filipino Americans (FAs) are one of the fastest-growing Asian American groups in the United States (1). Filipino immigration has filled labor shortages in agriculture, the military, and nursing, and FAs are overrepresented in these important workforces (2). It is important that FAs who fill these jobs are healthy.

FA engagement in health promotion is critical because FAs continue to have a higher prevalence of chronic diseases than whites, blacks, and other Asian groups (3–6). FAs have a high prevalence of and high mortality rates due to cardiovascular disease (CVD) and diabetes (7–11). Research shows that FAs are 18% more likely to have hypertension than other Asians or whites (4). An estimated 28% of FAs have CVD (6), and FAs are diagnosed with diabetes 8.4 years earlier than whites (11).

FAs also report a high prevalence of behavioral risk factors such as obesity, smoking, binge drinking, and physical inactivity (3–6). A 2007 needs assessment of FAs in New York indicated that CVD is a primary health concern, noting the lack of healthful eating, exercise, and health care access in this population (4,5,9,12). Researchers reported frustration on behalf of FAs over the lack of culturally appropriate health resources for FAs (12).

Cultural tailoring of interventions can be effective in reducing health disparities by attracting underserved populations to health promotion programs and improving their outcomes (13,14). Culturally tailored interventions significantly improve cancer screening rates, diet, and readiness to quit smoking (13). Such interventions incorporate culture on the surface level and on deep levels (15). Specifically, educational materials, communication channels, settings, staff, and recruitment strategies reflect the target population (surface level), and the intervention builds on cultural values (deep level). Spirituality, family, upward mobility, caring orientation, connection to the Philippines, arts, food, and life celebrations are integral Filipino values (16).

The purpose of this systematic review was to assess interventions that increase FA participation in CVD prevention programs, examine intervention effectiveness, and identify key cultural components of tailored interventions.

## Methods

### Data sources

PubMed MEDLINE, CINAHL, and Sociologic Abstracts were searched for peer-reviewed studies and dissertations conducted in the United States from 2004 through 2016 by using combinations of the following search terms on FAs: (Philippines/ethnology, Filipin*, Asian American, culture), interventions (health promotion, preventive health services, prevention and control), and conditions (cardiovascular disease, diabetes, cerebrovascular disease). Citations of relevant articles were reviewed to capture additional studies. This review followed Preferred Reporting Items for Systematic Reviews and Meta-Analyses (PRISMA) guidelines (17).

### Study selection

One reviewer (J.D.) assessed the relevance of articles and retained those that reported on interventions that aimed to prevent or manage CVD and risk factors, recruited at least one FA group, and reported how the intervention was or could be tailored to FAs. For comparative studies, the comparators were defined as the group to which the intervention was not delivered or the non-FA ethnic groups to which the intervention was targeted. Outcomes included any chronic disease indicators and changes in perceptions around chronic disease. To understand the cultural tailoring or adaptation of included interventions, we incorporated qualitative studies that provided strategies to tailor programs to FA communities.

### Data extraction

We examined the presence of cultural components, evaluation methods, and sustainability efforts of each intervention. Data were abstracted on the proportion of FAs included in the study, study type, intervention focus, cultural components, evaluation methods and findings, and implementation and sustainability issues (18). Articles were reviewed to determine the presence of core Filipino values of spirituality, family, upward social mobility, caring orientation, connection to the Philippines, arts, food, and life celebrations (16).

The quality of each quantitative study was assessed using a modified version of the Community Preventive Task Force’s assessment tool (19). Each study was scored based on 9 domains, including intervention description, sampling frame, eligibility criteria, population sampling, intervention exposure, valid and reliable outcome measures, appropriate statistical analysis, participant completion, and controlling for confounders. Scores ranged from 0 to 9 based on the number of criteria met and were rated as good (8 or 9 criteria met), fair (5–7 criteria met), or limited (<5 criteria met) (19).

Qualitative studies were appraised using a modified form of Schou and colleagues’ assessment tool for qualitative research (20). Each study was rated based on the following 5 criteria: formal requirements of conducting research, credibility of study design, transferability of findings, dependability of data analysis, and confirmability of research findings. Each domain was rated on a scale of 1 (complete disagreement) to 4 (complete agreement). Scores ranged from 0 to 20 on the basis of the extent to which the criteria were met. Total scores were calculated, and each study was rated as “recommended” (scores ≥15), “recommended with reservations” (scores of 10–15), or “not recommended” (scores <10) (20).

Intervention efficacy was assessed by using Spencer and colleagues’ framework for planning and improving evidence-based practices (21). This framework lists questions to pose about an intervention’s public health impact and the strength of the evidence in 5 areas: 1) program effectiveness, 2) potential intervention reach, 3) implementation feasibility, 4) sustainability, and 5) applicability in various contexts (transferability). A program’s effectiveness was determined by achievement of outcomes, public health significance, and magnitude of effect. Reach was assessed by proportion of eligible participants affected, potential to reach participants, and representativeness of groups. Feasibility was evaluated by the degree of implementation barriers, facilitators, and resource needs encountered when implementing the intervention. Sustainability was determined by the degree of integration into existing systems, outcomes, and resources required to sustain the practice. Transferability was assessed by the ability to be replicated in other settings, degree of adaptation needed for other populations, and comparative effectiveness to other studies. Each area was rated from 1 (low) to 4 (high). Scores then were averaged across the 5 areas to create a total impact score, and each article was rated as low (scores <2), moderate (scores of 2 or 3), or high (scores >3). Each program was plotted at the intersection of its impact scores from the quality assessments (*x* axis) and its mean impact score (*y* axis) (21).

## Results

### Article selection

**Figure 1:**
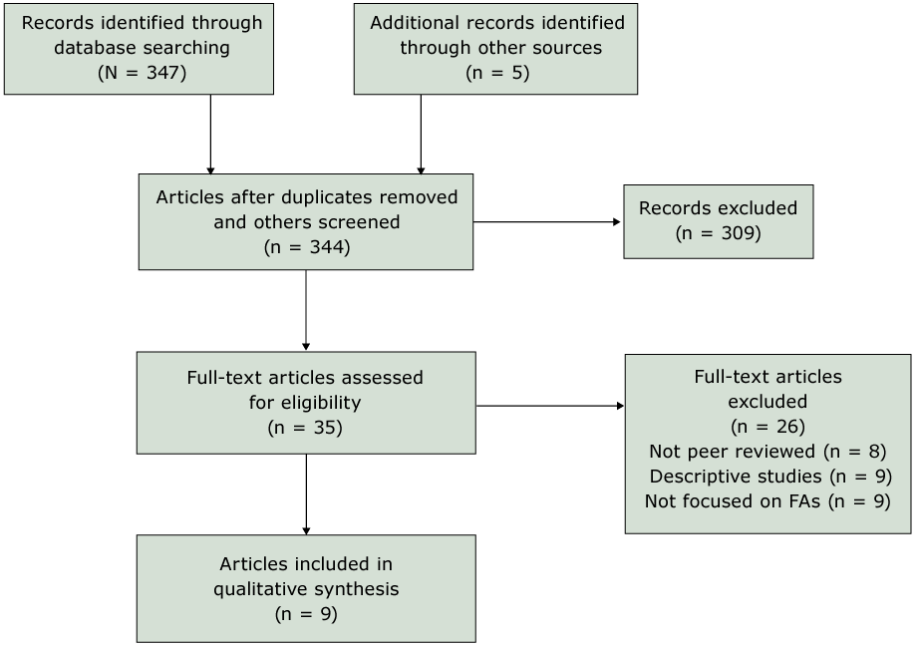
Preferred Reporting Items for Systematic Reviews and Meta-Analyses (PRISMA) flowchart for article selection, review of articles on increasing Filipino Amercian participation in cardiovascular disease prevention programs, United States, 2004–2016.

A total of 347 articles were identified (Figure 1). Duplicates (n = 8) and nonrelevant articles based on title and abstract (n = 309) were removed. One reviewer read the remaining 30 in full, and 5 additional articles were found through manual review of reference lists. Upon application of inclusion and exclusion criteria, 26 of the 35 were excluded: 9 were descriptive studies, 9 did not target the FA community, and 8 were not peer reviewed articles. The remaining 9 articles reporting on 7 interventions were included.

### Study descriptions

Eight articles reported quantitative data from 7 interventions (22–29). The ninth article reported qualitative findings from focus groups and did not test an intervention (30).

Of the 7 interventions, 3 were tested through randomized controlled trials (23,24,27,28), and 4 were single-group pretest–posttest studies (22,25,26,29). Four interventions were conducted in Hawai‘i (22–26), 2 in California (27,28), and one in New Jersey/New York (29) (Table 1). Two of the 7 interventions focused on diabetes and CVD prevention (23,24,27), 2 on CVD risk reduction (22,29), one on general chronic disease prevention (28), one on diabetes management (26), and one on chronic disease management (25). Both diabetes prevention interventions combined aspects of the Centers for Disease Control and Prevention’s (CDC’s) Diabetes Prevention Program and the National Heart, Lung, and Blood Institute’s (NHLBI) Healthy Heart, Healthy Family (HHHF) curricula (23,24,27). For articles focused solely on CVD risk reduction, researchers tested the HHHF curriculum tailored for FAs (22,29). Articles published by Tomioka and colleagues tested evidence-based curricula developed by Stanford University for management of chronic conditions (25) and diabetes (26). Dirige and colleagues tested a non-evidence–based curriculum for their intervention (28).

**Table 1:** Description of Articles (N = 9) Included in Analysis, Review of Studies on Increasing Filipino American Participation in Cardiovascular Disease Prevention Programs, United States, 2004–2016

Source	Location	Study Design	Intervention Focus	Health Education Curricula	Intervention Duration	Class Length	Sample Size of FAs, No.
Bender et al, 2016 (27)	San Francisco, California	RCT with waitlist control	Diabetes prevention	DPP plus NHLBI Healthy Heart, Healthy Family for FAs (PilAm Go4Health)	6 Months (3-month intervention plus 3-month maintenance)	7 In-person visits total	45
Fernandes et al, 2012 (22)	Honolulu, Hawai‘i	One group pre–post test	CVD risk reduction	NHLBI Healthy Heart, Healthy Family for FAs	11 Weeks plus 1-year follow-up	2-Hour sessions (11 sessions total)	92
Inouye et al, 2014 (23); Leake et al, 2012 (24)	Honolulu, Hawai‘i	RCT with waitlist control	Diabetes prevention	DPP plus Healthy Heart, Healthy Family (Health is Wealth)	6 Weeks to 6 months	90-Minute sessions (8 sessions total)	40
Ursua et al, 2014 (29)	New Jersey and New York State	One group pre–post test	CVD risk reduction	NHLBI Healthy Heart, Healthy Family for FAs	4 Months	90-Minute sessions (4 sessions total)	39
Tomioka et al, 2012 (25)	Honolulu, Hawai‘i	One group pre–post test	Chronic condition management	Stanford CDSMP	6 Weeks	2.5-Hour sessions (6 sessions total)	160 (FA)
Dirige et al, 2013 (28)	San Diego County, California	RCT	Chronic disease prevention	Nutrition physical activity program (*Siglang Buhay*)	18 Months	Not reported	255
Tomioka et al, 2014 (26)	Honolulu, Hawai‘i	One group pre–post test	Diabetes self-management	Stanford DSMP	6 Weeks	2.5-Hour sessions (6 sessions total)	82 (FA)
Finucane and McMullen, 2008 (30)	Honolulu, Hawai‘i	Qualitative	Diabetes self-management	Diabetes self-management education	—	—	—

Abbreviations: — , not applicable; CDSMP, chronic disease self-management program; CVD, cardiovascular disease; DPP, diabetes prevention program; DSMP, diabetes self-management program; FA, Filipino American; NHLBI, National Heart, Lung, and Blood Institute; RCT, randomized controlled trial.

Delivery of curricula varied across interventions. For articles describing the combined CDC and NHLBI curricula, Inouye et al and Leake et al (23,24) offered participants a choice to complete all 8 sessions in the 6-month intervention period, with some finishing the intervention in as little as 6 weeks. In contrast, Bender et al (27) delivered their curriculum virtually in conjunction with 7 in-person visits for intervention education, coaching, and support. In studies testing an NHLBI curriculum, intervention intensity ranged from 4 monthly sessions with 6 hours of education total (29) to 11 weekly sessions, totaling 22 hours of education (22). The sample size of FAs was small, with 6 of the 8 studies reporting samples of fewer than 100 FA participants (22–27,29). Dirige and colleagues’ study yielded the highest participation, with 255 FAs recruited through community-based leaders who organized activities and educational sessions to support healthy eating and physical activity (28).

### Recruitment strategies

Of the 7 interventions, only 2 did not use word of mouth from friends, family, and community leaders as the primary recruitment method for interventions (23–27,29). Of those that did not use word of mouth advertising, Fernandes and colleagues relied on direct physician referrals to the program (22), and Dirige and colleagues sent mailers to zip codes where FAs resided (28). Community events also were popular methods of recruitment across the interventions. Events involved varied activities such as health screenings (23–25,29), social groups and cultural events (27–29), and church activities (23–25,29). Furthermore, Tomioka and colleagues posted advertisements in newspapers and offered an informational session to recruit interested participants (25,26).

### Cultural tailoring and Filipino values

Of all the Filipino cultural values described by Guerrero and colleagues (16), the most prevalent was the incorporation of traditional Filipino foods (Table 2) (22–24,26–29). Each intervention integrated Filipino foods differently. Two offered replacements to modify traditional foods, for example, suggesting grilled fish instead of fried fish (27,28). However, most interventions aimed to increase awareness of the nutritional value (or lack thereof) of common Filipino foods through pictures (22) or verbally by using Filipino words for food (eg, *chicharon* instead of pork rinds) (23–26,29). Evidence from the single qualitative study underscored the importance of tailoring education around diet modification while respecting the significance that food plays in the Filipino culture (30).

**Table 2:** Incorporation of Filipino Values in Interventions and Associated Outcomes, Review of Studies on Increasing Filipino American Participation in Cardiovascular Disease Prevention Programs, United States, 2004–2016^a^

Characteristic	Study						
	Ursua et al, 2014 (29)	Fernandes et al, 2012 (22)	Bender et al, 2016 (27)	Inouye et al, 2014 (23); Leake et al, 2012 (24)	Tomioka et al, 2012 (25)	Tomioka et al, 2014 (26)	Dirige et al, 2013 (28)
**Tailoring for Filipino Americans**							
Spirituality	X			X	X		
Family	X	X	X	X			
Upward social mobility	X	X	X		X	X	
Caring and social relationships	X	X	X	X	X	X	X
Country	X	X	X	X			
Arts	X		X				X
Food	X	X	X	X	X	X	X
Life events	X	X			X	X	X
**Outcomes**							
Clinical	Improvements in BP and BMI	NS	NA	Improvements in BMI, weight, and waist circumference	NA	Improvements in BMI, HbA1c, cholesterol, and BP	NA
CVD knowledge	+	+	NA	NA	NA	NA	NA
Behavior	Improvements in health behaviors	Improvements in diet and self-management	NA	NA	Improvements in doctor communication and self-management	Improvements in health behaviors, self-monitoring, self-rated health, and coping	+
Satisfaction	+	+	NA	+	+	NA	NA

Abbreviations: BMI, body mass index; BP, blood pressure; HbA1c, hemoglobin A1c; NA, not applicable (final results still pending intervention completion); NS, not significant.

a “X” indicates that the component was included in the intervention; “+” indicates overall improvement.

All 7 interventions incorporated the cultural value of caring and maintaining social relationships during the course of the intervention (22–29). This included the use of Filipino staff to foster social support through group discussions and activities. Ursua and colleagues partnered with local community-based organizations and churches to help sponsor health screenings (29). Bender and colleagues established a private social media page to promote social support through a virtual platform (27). Dirige and colleagues trained volunteer Filipino club leaders to organize health promotion activities such as walking clubs and instituting healthy food potluck policies for their groups (28). Similarly, Tomioka and colleagues reported that there was “ethnic concordance” between participants and leaders, which facilitated the recruitment and retention of FA participants (25,26).

The incorporation of Filipino staff with shared language, culture, and life experiences helped to build rapport and trust among participants. Furthermore, the inclusion of Filipino staff who were accessible to participants was vital to maintaining these relationships. Flexible scheduling and follow-up were important aspects to maintain trust and participation in the various programming. Ursua and colleagues noted that their Filipino staff was available on nights and weekends to accommodate the participants’ work and family schedules (29). Inouye et al and Leake et al echoed the value of flexible scheduling to retain participants in their program (23,24). Finucane and McMullen also noted the importance of working together for the benefit of all (*bayanihan*), a common traditional value among Filipinos, through social support and storytelling (30).

Four of the 7 interventions noted the importance of involving family members in the intervention (22–24,27,29). Most interventions encouraged participants to invite their family members to join the educational sessions (22–24,27,29). One intervention encouraged participants to role-play with family members by using the scenarios presented as part of the curriculum (23,24). Finucane and McMullen emphasized the struggle FAs often face managing their chronic conditions while not burdening their family members (30).

Upward social mobility, which referred to career advancement through the pursuit of higher education, was seen in 5 interventions (22,25–27,29). This value was exemplified through the inclusion of graduation ceremonies that conferred a certificate of completion following the intervention. Tomioka and colleagues noted that participants were proud “to have a certificate from the Stanford program” (26). Ursua and colleagues noted that these graduation events allowed participants to share their successes and celebrate the progress that was made (29). Similarly, other life celebrations were incorporated in 5 interventions (22,25,26,28,29). Reunions and monthly celebrations also served as a mechanism to capture follow-up data (22,25,26).

Three interventions addressed the value related to a connection to the Philippines through the expression of traditional values that were embodied within the interventions (22–24,27,29). Ursua and colleagues (29) and Bender and colleagues (27) framed CVD education in the context of Filipino history and culture. Participants of the Hawai‘i-based diabetes intervention reported that classes were reminiscent of a visit to the Philippines (23,24). Fernandes and colleagues reported that the Filipino staff instilled the values of togetherness (*pakkikisama*), community spirit (*bayanihan*), and obligation and reciprocity (*utang-na-loob*) within the program (22).

Spirituality and the arts were the least incorporated values; spirituality was mentioned in 3 interventions (23–25,29) and the arts in 3 interventions (27–29). Spirituality was integrated through opening prayers (25) or engagement of local pastors to assist in the recruitment of participants or reinforcement of health messages (23,24,29). In their qualitative study, Finucane and McMullen noted that spirituality plays a significant role in coping with and understanding of illness (30). These authors also noted the importance of the concept of *bahala na*, a traditionally fatalistic view of illness, which may contradict the concepts of self-management often recommended in chronic disease management. The Filipino arts were incorporated through encouragement of physical activity by dancing (27,28). One intervention incorporated singing and dancing marketed as a social event for screening, education, and recruitment into the intervention (29). The 3 articles that incorporated Filipino arts were all located outside of Hawai‘i.

### Intervention outcomes

Intervention outcomes fell into 4 categories: clinical, CVD knowledge, behavioral, and participant satisfaction (Table 2). Only 4 articles measured and reported clinical outcomes, whereas others reported behavioral outcomes, participant satisfaction, or both. Of those reporting clinical outcomes, Ursua and colleagues’ HHHF intervention, which incorporated all the cultural values, reported the most positive outcomes across all categories (29). Fernandes and colleagues implemented the same curriculum and reported similar satisfaction findings but did not realize improvements in clinical outcomes (22). By using the CDC and NHLBI curricula, Inouye and colleagues observed decreased weight, waist circumference, and BMI in addition to high participant satisfaction and attendance (23,24). Tomioka and colleagues found significant decreases in BMI, improved HbA1c, and lower cholesterol and blood pressure levels (26). Bender and colleagues showed promising findings; however, final results are still pending intervention completion (27). In general, improvements in health behaviors of physical activity and diet were seen in all interventions. Satisfaction and retention remained high among those interventions that captured that information. All interventions showed positive findings across various outcome measures.

### Quality assessment and intervention efficacy

Of the 7 quantitative studies, 3 were rated as good quality (27–29) and 4 were of fair quality (22–25,26) (Table 3). The single qualitative study was rated as a recommended study with a total score of 18 of 20 (30).

**Table 3:** Quality Assessment and Intervention Efficacy, Review of Studies on Increasing Filipino American Participation in Cardiovascular Disease Prevention Programs, United States, 2004–2016

Source	QA Score^a^	Effectiveness^b^	Reach^b^	Feasibility^b^	Sustainability^b^	Transferability^b^	Total Impact Score^c^
Dirige et al, 2013 (28)	8	3.7	3.3	3.0	2.7	1.7	2.9
Ursua et al, 2014 (29)	8	3.0	3.0	2.7	2.0	3.3	2.8
Bender et al, 2016 (27)	8	1.3	3.7	1.7	1.3	1.3	1.9
Fernandes et al, 2012 (22)	7	2.7	2.3	2.7	1.7	3.3	2.5
Tomioka et al, 2012 (25)	7	3.3	3.0	2.7	2.7	3.7	3.2
Tomioka et al, 2014 (26)	7	3.7	3.0	2.7	2.7	3.7	3.1
Inouye et al, 2014 (23); Leake et al, 2012 (24)	6	2.7	2.3	3.0	1.7	2.3	2.4

a Quality assessment (QA) score is the total number (of 9) of quality criteria met by study. Total scores translated as <5 = limited quality; 5–7 = fair quality; and 8–9 = good quality.

b Effectiveness, reach, feasibility, sustainability, and transferability, scored from 1 = low to 4 = high.

c Total impact score is the mean of effectiveness, reach, feasibility, sustainability, and transferability scores for the intervention. Total scores translated as <2 = low impact; 2–3 = moderate impact; >3 = high impact.

**Figure 2:**
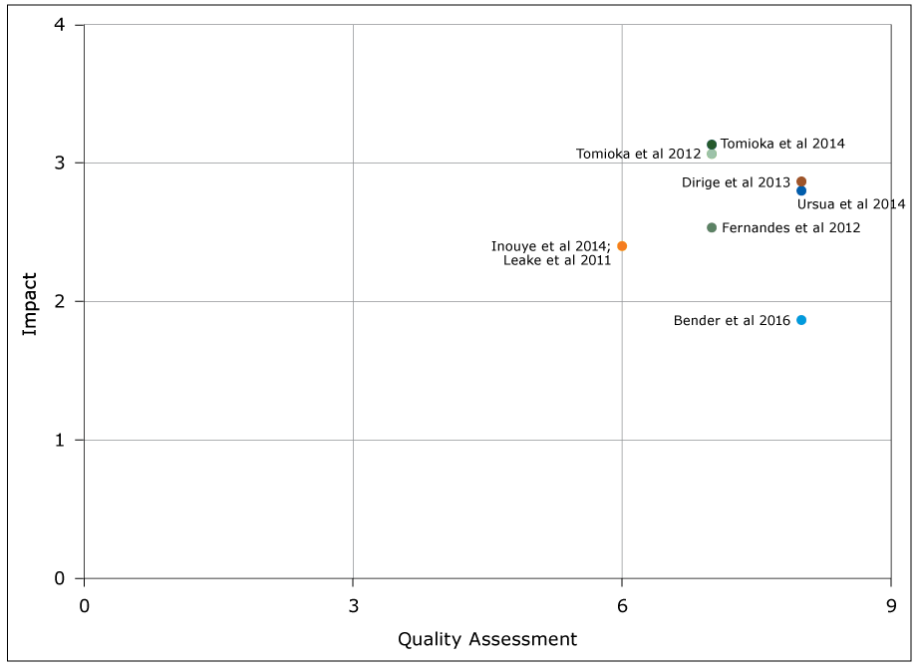
Graph of intervention efficacy according to the Spencer grid (21), review of articles on increasing Filipino Amercian participation in cardiovascular disease prevention programs, United States, 2004–2016. The quality assessment score is the total number of quality criteria of 9 total that were met by the study (<5 = limited quality, 5–7 = fair quality, 8–9 = good quality). The total impact score is the mean of effectiveness, reach, feasibility, sustainability, and transferability scores for the intervention (<2 = low impact, 2–3 = moderate impact, >3 = high impact).

In terms of intervention effectiveness, only 2 articles scored within the high range (26,28). Five of the 7 interventions had moderate reach (22–26,29). All but one article described moderately feasible interventions (27). Only 3 of the articles described interventions that were scored as moderately sustainable (25,26,28). Four of the interventions scored high transferability (22,25,26,29). Summing these scores, 4 interventions had moderate impact (22–24,28,29), and 2 were high-impact interventions (25,26). The impact score was lowest for the intervention for which final results were not available (27). Plotting these scores on the Spencer grid (21)(Figure 2) showed that interventions reported by Tomioka and colleagues (25,26) had the most impact, but studies by Dirige and colleagues (28) and Ursua and colleagues (29) were higher quality. All the studies were either leading or promising practices.

## Discussion

We identified 9 articles reporting on 7 interventions focused on CVD prevention among FAs in this systematic review. Most of the studies were conducted in Hawai‘i, and 3 were conducted in the continental United States. All but 2 articles involved a 100% FA sample. All but one study used evidence-based curricula, although implementation varied across sites. All but 2 interventions used word-of-mouth advertising to increase participation.

In terms of Filipino cultural values, food, caring and social relationships, and family were prevalent aspects across interventions tailored for FAs. The cultural significance of food among FAs was evidenced by its presence in all interventions. Researchers have noted that sharing a meal is a way to foster relationships and pay homage to the past. Rejection of food when offered, even for health reasons, may put a strain on social relationships (5,27,30). This poses a challenge to many FAs who must change their diet to prevent or delay the onset of CVD or other chronic conditions. FA-tailored interventions should involve families to support changes to their lifestyle and diet. Studies suggest that life celebrations during which families and friends gather and share meals may serve as opportunities for health interventions.

Dalusung-Angosta echoed the importance of considering social and cultural values when delivering CVD prevention education (31). In her study of 120 FAs recruited from 3 primary care clinics in Las Vegas, Nevada, she observed that FAs were knowledgeable about CVD, but knowledge was not enough to prevent CVD among FAs. Furthermore, Feldman and Sills suggested that a person’s hope was a significant predictor of their behaviors related to CVD prevention (32). Asians with high hope and CVD knowledge were more likely to lower their salt and fat intake, proactively seek CVD information, and visit their physician more often (32). This finding further emphasizes the importance of caring and social relationships in nurturing a sense of hope among FAs managing their health.

Only 3 interventions integrated aspects of spirituality (23–25,29) and 3 integrated the arts (27–29). Religion plays a large role in FA culture, with nearly all FAs identifying either as Catholic or Protestant (33). Spirituality provides meaning to many FAs, shaping their interpretation of illness, self-management of health conditions, and relationships with others (30,34,35). Similarly, FA participation in cultural arts and reinforcement through mass media may encourage healthy behaviors. A small study of FA women in San Francisco reported dancing as the most prevalent form of physical activity (36). In interviews with community leaders and FAs in Hawai‘i, Pobutsky and colleagues noted the opportunity to leverage Filipino television programs and advertisements to reinforce the importance of taking care of their health to live a long life with their family (35). Furthermore, an assessment of FAs in California noted that Filipino radio, newspapers, and magazines are a source of health information for FAs, after medical providers (34). Hence, spirituality and arts may be avenues for health interventions.

We found only 4 articles that reported on HHHF, a curriculum tailored specifically for FAs, and only 2 articles reported using the curriculum in its entirety. A review of the NHLBI website indicated that this curriculum was tested in another community in Hawai‘i. Although this site reported high participant satisfaction and increased referrals to health care professionals, it did not report outcome data, making it difficult to ascertain the intervention efficacy (37). Research consistently reports high prevalence of hypertension among FAs; however, culturally tailored programs like HHHF have not been implemented widely throughout these communities.

Although intervention efficacy across the studies looked promising, the articles varied in outcome measures and intervention delivery. Therefore, drawing conclusions from the intervention results was challenging. Even across the same curriculum, the variability in intervention delivery and intensity were not comparable enough to infer essential cultural values to include in the curriculum.

### Limitations

Our study has several limitations. Publication bias is a possible limitation, because studies with negative findings may not move forward to publication. Although 3 databases were searched, relevant literature may be available elsewhere. The variability across interventions concerning duration and total sessions made it difficult to compare findings across studies. Using the Spencer et al framework helped mitigate some of the challenges in assessing the efficacy of interventions among the articles (21). Another limitation is the lack of information on generational status. Only 2 articles (22,30) captured data related to immigration or length of US residence. Research shows that one’s generational status may influence self-care behaviors (30). Capturing this information may have elucidated the degree of cultural tailoring needed to address barriers to healthy behaviors.

### Implications for practice and research

Given the high prevalence of CVD in FA populations, tailored interventions rooted in Filipino cultural values are vital to address this known health disparity. Future interventions engaging FAs should use word-of-mouth recruitment strategies. Health education curricula should acknowledge the cultural significance of food, encourage family participation in sessions, and foster a supportive environment to build relationships among participants. Future research should aim to include larger samples of FAs across the United States with long-term follow-up and should account for acculturation. Research also should identify and collect standardized metrics to demonstrate the efficacy of the interventions and data on participant satisfaction.
